# Emotional intelligence in teachers of educational institutions in the department of Magdalena

**DOI:** 10.1192/j.eurpsy.2024.1716

**Published:** 2024-08-27

**Authors:** K. L. Perez Correa, J. Viloria Escobar, L. P. Pedraza Àlvarez, M. Egea Lavalle

**Affiliations:** ^1^Magdalena, Universidad Cooperativa de Colombia, Santa Marta; ^2^Magdalena, Instituto Nacional de Formación Técnica Profesional Humberto Velásquez García, Cienaga; ^3^Magdalena, Universidad del Magdalena, Santa Marta, Colombia

## Abstract

**Introduction:**

Psychosocial and mental health-related variables are crucial determinants of individuals’ lives in society and their roles within organizations, especially in educational institutions that are characterized by social complexities. In this regard, this research aims to determine the levels of emotional intelligence among teachers in educational institutions in the Department of Magdalena.

**Objectives:**

Determine the levels of emotional intelligence among teachers in educational institutions in the Department of Magdalena

**Methods:**

Methodologically, it is situated within the empirical-analytical paradigm with a quantitative approach, using the descriptive method. A convenience sample of 179 teachers was used, and the TMMS-24 questionnaire was administered.

**Results:**

The results revealed that 37.7% of the teachers completely agree, and 30.9% strongly agree with the statement that they pay a lot of attention to their feelings. On the other hand, only 12.2% somewhat agree, and 1.2% strongly disagree with the statement that they normally worry about what they feel.

Additionally, 33.9% agree with the statement that they usually take time to think about their emotions, while 25.6% somewhat agree, and only 1.2% strongly disagree with the statement that it is worth paying attention to their emotions and mood. Furthermore, 6.4% agree, and 5.2% strongly agree with the statement that they let their feelings affect their thoughts.

As for thinking about their mood constantly, 16.7% strongly disagree, and 39.1% somewhat agree. Moreover, 6.9% strongly disagree, and 21.4% somewhat agree with the statement that they pay a lot of attention to what they feel.

Only 1.1% strongly disagree with being able to frequently define their feelings. and only 1.7% strongly disagree, and 10.2% somewhat agree with the expression “I often become aware of my feelings in different situations.” 34.1% strongly agree, and 26.6% completely agree with the statement “I can always tell how I feel.” Finally, 5.1% strongly disagree, and 19.4% somewhat agree with the statement “Sometimes I can tell what my emotions are.”

**Conclusions:**

In conclusion, the study emphasizes in the importance of teachers’ emotional intelligence and its potential impact on their performance and students’ learning outcomes. It also highlights the need for intervention strategies to strengthen this psychosocial variable in educational institutions in the Department of Magdalena.

**Disclosure of Interest:**

None Declared

